# Sun Stroke

**Published:** 1860-09

**Authors:** 


### SUN STROKE.

On this subject Dr. L. Ch. Boisliniere, Coroner of St. Louis county, Mo., publishes in the *St. Louis Medical and Surgical Journal*, for July, a report of seventy-two cases observed by him.

The necropsies in these cases revealed the following conditions:

*External Appearances.*—Marked lividity of the skin; neck and anterior part of chest became soon of a purple or blue color; in a few hours the abdomen was quite tympanitic, an abundant froth came out of the mouth and nostrils, resembling thick lather, mixed sometimes with a little blood. By pressing upon the chest, blood could be made to flow freely from the mouth and nostrils.

The lungs and heart were in every case seen to be more or less conjested; the right side of the heart and the pulmonary artery generally contained black and liquid blood; left side empty; on section, the lungs were found to contain an abundant quantity of frothy mucous, mixed with more or less arterialized blood. By moderate pressure on the chest, as above observed, this bloody froth could be made to run out freely from the mouth and nostrils. So characteristic was this appearance, that from its presence alone many post mortem examinations towards the end of the summer were dispensed with, the jury, before the writer, after a short explanation, being able to make a satisfactory verdict of death by sun stroke.

The brain was generally found normal; in a few cases only there was moderate congestion of the superior cerebral veins and of the sinuses. This the author accounts for by the difficulty the blood found in returning from the head to the thoracic organs, already full of venous blood. Occasionally the ventricles of the brain contained a little more serum than usual. This Dr. B. attributes to the obstacle about the heart and lungs, damming up the blood in the veins of the brain and its sinuses, and causing some of the serum to ooze out. This effusion of serum was often quite remarkable on the surface of the brain under the membranes, where it frequently assumed an opalescent appearance.

Liver and spleen were, as a rule, enlarged; the spleen particularly softened.

The author remarks that these appearances coincide with what has been observed by several distinguished writers, among whom are Andral, Russell, Gerhard, and Levick.

*Cause.*—The cause of sun-stroke, according to our author, sustained by the best authorities, without doubt, is a hot and rarified atmosphere—the want of oxygen; for the disease occurs very frequently in houses where, from some cause, the air has become rarified. Several of the deaths reported above by the writer, having taken place in low attic rooms, in kitchens or laundries, and in sugar refineries, as observed by Dr. H. H. Swift, (*New York Journal of Medicine*, vol. xiii, p. 45, 1854.) From these and other observations, Dr. B. concludes that rarified, or poorly oxygenated air, is the "*conditio sine qua non*" of sun stroke.

---

HOMŒOPATHY.—Like cures like. Sulphur comes from Vesuvius. Therefore it is good for eruptions.—*Punch.*

